# Idiopathic Eosinophilic Vasculitis: Case Presentation and Literature Review

**DOI:** 10.5041/RMMJ.10498

**Published:** 2023-04-30

**Authors:** Adi Broyde, Ori Elkayam

**Affiliations:** Rheumatology Department, Tel Aviv Sourasky Medical Center, Tel Aviv, Israel

**Keywords:** Digital ischemia, eosinophilic disorders, eosinophilic granulomatosis with polyangiitis, eosinophilic vasculitis, hypereosinophilic syndrome, mepolizumab

## Abstract

**Objective:**

Idiopathic eosinophilic vasculitis has been described in previous case series as a possible manifestation of hypereosinophilic syndrome (HES) in asthma-free patients. A rare disease, it can be classified as an eosinophilic-rich, necrotizing, systemic form of vasculitis that affects vessels of various sizes in these patients. This report shares our experience with the treatment of a patient with eosinophilic vasculitis.

**Case Presentation:**

We present the case of a 45-year-old man who suffered from idiopathic HES manifesting as digital ulcers and peripheral ischemia of both the upper and lower limbs without the involvement of other systems. Diagnosis was made after excluding the primary and secondary causes of eosinophilia. The patient responded well to both corticosteroids and mepolizumab, an interleukin-5 inhibitor, as a corticosteroid-sparing therapy.

**Conclusion:**

Our case of HES-associated vasculitis in an asthma-free patient supports previous reports describing this rare diagnosis of idiopathic eosinophilic vasculitis in recent years. We describe a good response to mepolizumab (interleukin-5 inhibitor) in our patient.

## INTRODUCTION

Idiopathic eosinophilic vasculitis has been previously described as a possible manifestation of hypereosinophilic syndrome (HES) in asthma-free patients.^1^ An eosinophilic-rich, necrotizing, systemic form of vasculitis, it affects vessels of various sizes in asthma-free patients. A recent case series described 117 patients with this form of vasculitis.^1^ However, only few of those described had ischemic manifestations that resembled thromboangiitis obliterans (TAO) as in the case we describe herein.

## CASE PRESENTATION

**Figure 1:**
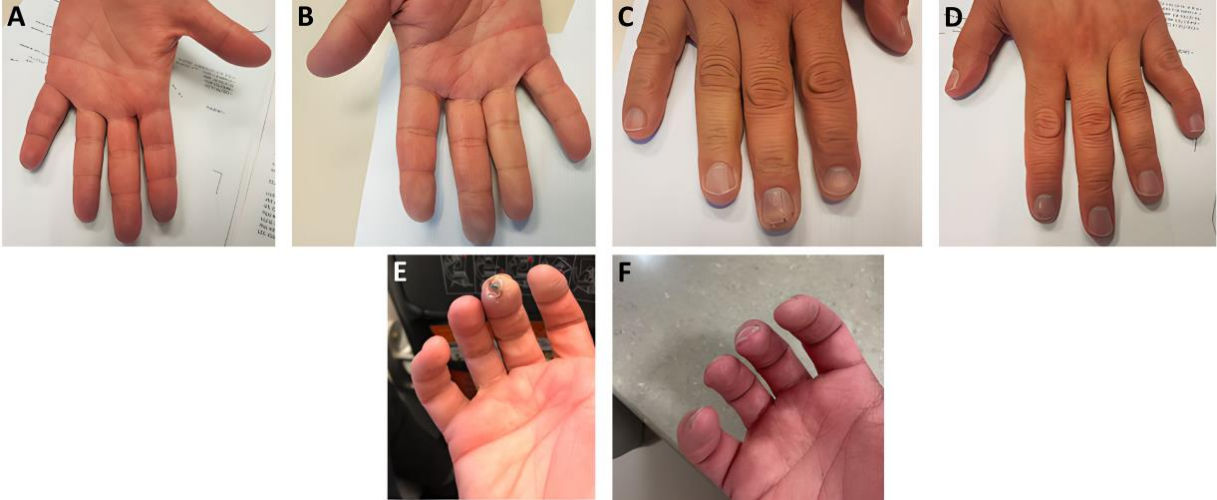
The Patient’s Hands with Ischemic Manifestations. **A, B, C, and D:** Bilateral finger pallor, cyanosis, and splinter hemorrhage. **E:** A digital ulcer that developed a few days after the initial presentation. **F:** The same finger a few months after the patient received steroids and mepolizumab therapy. Image quality enhanced for publication using AI Image Upscaler (Zyro.com).

Our patient was a 45-year-old man who presented with bilateral finger pallor, cyanosis, splinter hemorrhages (Figure 1A–D), and a digital ulcer (Figure 1E) that caused severe pain. He also complained of paresthesia of the toes and intermittent claudication of lower limbs. His symptoms had begun 2 months earlier.

The patient’s medical history included ulcerative colitis, diagnosed 9 years earlier and in remission. His regular medications included 5-aminosalicylic acid alone. He had no history of smoking. On physical examination his distal fingers were cold, mildly cyanotic, and he had a digital ulcer on the third right finger, causing severe pain. Mild lower leg edema was present and peripheral pulse examination revealed intact ulnar and radial pulses; however, pulses were absent in the dorsalis pedis. Initial bedside Doppler of only the upper limbs showed intact radial pulses with no Doppler signal over the palmar arches.

### Lab Work, Imaging, and Other Test Results

A complete blood count revealed an eosinophil count of 4,200 cells/μL, white blood cell count 11,000 cells/μL, and hemoglobin 15 g/dL. C-reactive protein (CRP) was mildly elevated (15 mg/L). Creatinine, creatinine-phosphokinase (CPK), liver functions, troponin, and urinalysis were all within normal ranges. An infection workup was negative and included blood cultures and the following tests: human immunodeficiency virus, venereal disease reference lab, *Treponema pallidum* hemagglutination, hepatitis B and C viruses, cytomegalovirus, Epstein–Barr virus, *Strongyloides*, *Toxocara*, and stool tests for ova and parasites.

Bone marrow biopsy showed hypereosinophilia with no other pathology.

Further workup for hematologic genetic abnormalities and clonal causes of eosinophilia was performed in the hematologic department of our hospital according to the World Health Organization (WHO) guidance for the diagnosis of primary (clonal) eosinophilic disorders^2^ and was negative. Most immune serology was negative, including antinuclear antibody, rheumatoid factor, anti-phospholipid panel, and cryoglobulins. Complement (C3, C4) and immunoglobulins were within normal range. However, conflicting results were received for the perinuclear anti-neutrophilic cytoplasmic antibodies (P-ANCA) serology: the test results from the local health maintenance organization were significantly positive, whereas our hospital laboratory’s results were negative.

To determine the “true” test results we performed a thorough investigation into the testing methodology used. Our findings revealed that the local health maintenance organization had not followed the accepted antineutrophil cytoplasmic antibodies (ANCA) assay guidelines. When we repeated the ANCA (immunofluorescence assay) tests in the hospital laboratory they were negative, as well as the anti-myeloperoxidase (MPO) and proteinase-3 (PR3) tests; atypical ANCA was positive. These test results all led to the conclusion that the P-ANCA result was a false positive in that patient.

Echocardiography and computed tomography angiography (CTA) of the chest and abdomen showed no pathology. Electromyography showed bilateral carpal tunnel syndrome and mild sensory polyneuropathy of the lower limbs.

### Summary of Findings

To summarize, we had a young patient with peripheral ischemic manifestations. Other findings included peripheral sensory polyneuropathy, marked hypereosinophilia, and mildly elevated CRP. Echo-cardiography and CTA of the chest and abdomen showed no pathology.

### Treatment and Clinical Course

In the days following presentation at our hospital, the patient experienced an exacerbation of finger cyanosis in both hands and the digital ulcer had worsened, with a small gangrenous area at the fingertip. The patient was treated with aspirin and i.v. prostacyclin for his ischemic manifestations; he was also empirically treated for a possible parasitic infection with a course of albendazole. Previous treatment with aminosalicylates (5-aminosalicylic acid) was stopped for the possibility of drug-induced hypereosinophilia. None of these treatments affected the patient’s condition.

We then started treatment with prednisone 1 mg/ kg. Significant clinical and laboratory improvement was seen within a few days. The patient reported a significant improvement in the degree of digital pallor and in the intermittent claudication symptoms.

On physical examination the patient’s distal pulses of the lower limbs were now palpable. His eosinophil level dropped from >3,000 cells/μL to 500 cells/μL. He was discharged from hospital and continued follow-up and further workup as an outpatient in our clinic. At this time an arterial Doppler of the lower limbs revealed ischemia of the posterior right tibialis artery. A CTA of the lower limbs revealed bilateral arterial occlusion of the anterior and posterior tibialis arteries without distal reperfusion. These arteries were completely occluded without distal reperfusion—implying a thrombosis. The proxymal part of the popliteal artery was intact as well as the aorta and its branches. There were no signs of atherosclerosis.

**Figure 2:**
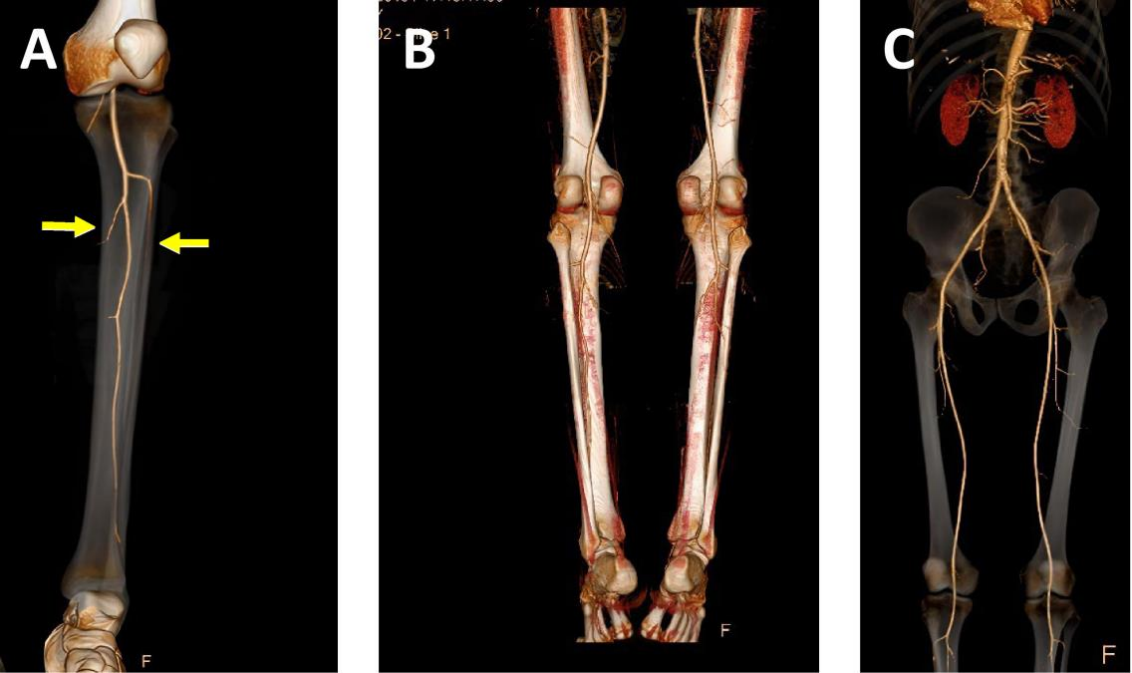
Computed Tomography Angiography of Thromboangiitis Obliterans (TAO)–like Vasculopathy. The patient’s CTA revealed severe vasculopathy of the lower legs with bilateral arterial occlusion of the anterior and posterior tibialis (arrows), without distal reperfusion **(A)**. There were no signs of atherosclerosis, and the proximal vessels were normal **(B, C)**. These imaging findings resembled TAO. Image quality enhanced for publication using AI Image Upscaler (Zyro.com).

Figure 2 presents different views of the patient’s CTA, with findings that resemble thromboangiitis obliterans (TAO). However, our patient was a non-smoker and his clinical and laboratory findings were not typical of TAO.

All of the patient’s symptoms, as well as the eosinophil and CRP levels, improved with steroid treatment. However, symptoms recurred when his prednisone dose was tapered below 40 mg/day. We recommended steroid-sparing therapy with cyclophosphamide. However, the patient refused this medication since the coronavirus 2019 pandemic had just begun and immunosuppression was more threatening than ever. The patient was treated with azathioprine but had to stop after 2 months due to drug-induced liver enzyme elevation and the lack of a clinical response to this medication. Mepolizumab was started with an impressive effect (Figure 1F), allowing for complete steroid discontinuation 4 months later.

Eight months following treatment with mepolizumab, CTA of the lower limbs still showed no reperfusion of the occluded arteries, probably reflecting irreversible vascular occlusion in this patient. At the time of writing, after 2 years of treatment with mepolizumab, we report that the patient is asymptomatic and without eosinophilia.

## DISCUSSION

Hypereosinophilic syndrome is a group of rare diseases (approximately 0.4 cases per 100,000 persons) defined as persistent eosinophilia >1,500 cells/μL (the threshold of 1500 cells/μL is arbitrary) together with end-organ damage.^2^

The first step in diagnosing HES is to differentiate between the primary (clonal) and secondary (reactive) causes of eosinophilia. The most common secondary causes include: infections, allergic/drug reactions, collagen-vascular disease (e.g. eosinophilic granulomatosis with polyangiitis, granulomatosis with polyangiitis, or systemic lupus erythematosus), pulmonary eosinophilic diseases, allergic gastroenteritis, and metabolic conditions such as adrenal insufficiency.^3^–^5^ A primary cause should be ruled out by blood tests in conjunction with bone marrow morphology and cytogenetic, immunophenotypic, and molecular analyses. Additional laboratory testing (e.g. troponin T or I, anti-neutrophil antibodies, anti-neutrophil cytoplasmic antibodies) and imaging tests (e.g. chest X-ray, electrocardiogram, echocardiography, and a CT scan of the chest, abdomen, and pelvis) are guided by the patient’s history and examination.

In the presented case, classic eosinophilic granulomatosis with polyangiitis (EGPA) could not be diagnosed since the patient had no pulmonary or ear, nose, or throat involvement, no history of asthma, no clear evidence of neurologic involvement, and negative serology for ANCA.^6^ The primary manifestations in our patient were bilateral finger pallor and cyanosis, as well as a digital ulcer, which are uncommon in ANCA vasculitis.^7^,^8^ Hence, our patient’s symptoms were compatible with a diagnosis of idiopathic HES. In a literature review that sought for similar cases, we found many similarities to a case series of idiopathic eosinophilic vasculitis (EoV) in asthma-free patients, published by Lefèvre and colleagues at the French National Reference Center for Hypereosinophilic Syndromes.^1^ The authors described 117 cases of idiopathic EoV that manifested as an eosinophil-rich, necrotizing vasculitis, which differed from EGPA in that it was non-granulomatous, and occurred in asthma-free, ANCA-negative patients. Idiopathic EoV is a variable-vessel vasculitis, which is non-granulomatous and predominantly involves the small vessels but may also involve the medium and large vessels. The suggested pathophysiology is the direct toxicity of activated eosinophils and extracellular granules that may be responsible for vascular wall necrosis in small to medium-sized vessels, and the occurrence of dissected arteries and aneurysms in medium- to large-sized vessels.^1^ Other clinical aspects include normal or mildly elevated CRP without gastrointestinal or kidney involvement. Interestingly, Lefèvre et al. reported pruritus in more than two-thirds of cases, which may be a hallmark of EoV since pruritus is rarely observed in other types of systemic vasculitis.

Idiopathic EoV has a good prognosis and responds well to corticosteroids. However, around 60% of the French group’s patients relapsed after tapering off of steroids and needed long-term steroid-sparing immunosuppression therapy.^1^

The characteristics of patients in the large French case series included median age of 43, equal distribution between males and females, and a single organ involvement in most patients. Clinical manifestations including coronary arteritis (30%) led to cardiac death in many cases. Cutaneous involvement (29%) included purpura urticaria, pruritic erythema, and digital necrosis. Peripheral nervous involvement (15%) included mainly mononeuritis multiplex and symmetric distal polyneuropathy. Temporal arteritis was also a manifestation of EoV in 11% of patients; these cases differed from typical giant cell arteritis by a normal erythrocyte sedimentation rate in most patients and the absence of giant cells in histology. Central nervous system involvement (6%) was also found, manifested by arterial dissection, intracerebral hemorrhage, and enhancement of brain parenchyma. These cases of central nervous system involvement were diagnosed by brain biopsy. The French researchers also found deep vein thrombosis (7%) as a possible manifestation of EoV.

Another interesting clinical manifestation of EoV found by the French group was thromboangiitis obliterans (TAO)-like disease. This finding caught our attention since they described 12 cases presenting similar clinical manifestations to our patient. Their manifestations included Raynaud’s phenomenon with diminished distal pulses and digital necrosis. Imaging findings included thromboangiitis obliterans-like obstructions and widespread blood flow alterations in the distal arteries. In the French cohort, 50% of the patients with thromboangiitis obliterans-like findings were non-smokers, another commonality with our patient. An arterial or other organ biopsy confirmed vasculitis in most of their described patients.^1^

## CONCLUSION

In cases of eosinophilia and vasculitis, idiopathic EoV can be diagnosed in asthma-free patients after excluding eosinophilic granulomatosis with polyangiitis, and secondary and primary causes of HES.

Idiopathic EoV is an additional form of variable-vessel vasculitis, and it has been suggested that vasculitis be added as a defining feature of HES. Our case supports information on this rare manifestation which has been described in a previous case series, and demonstrates the good prognosis of this form of vasculitis when treated with corticosteroids and interleukin-5 inhibitors.

## Abbreviations

ANCA: antineutrophil cytoplasmic antibodies
CRP: C-reactive protein
HES: hypereosinophilic syndrome
TAO: thromboangiitis obliterans
WHO: World Health Organization

## References

[1] Lefèvre G, Leures A, Gibier JB (2020). “Idiopathic eosinophilic vasculitis”: another side of hypereosinophilic syndrome? A comprehensive analysis of 117 cases in asthma free patients. J Allergy Clin Immunol Pract.

[2] Shomali W, Gotlib J (2022). World Health Organization -defined eosinophilic disorders: 2022 update on diagnosis, risk stratification, and management. Am J Hematol.

[3] Ganeva M, Gancheva T, Lazarova R (2008). Carbamazepine-induced drug reaction with eosinophilia and systemic symptoms (DRESS) syndrome: report of four cases and brief review. Int J Dermatol.

[4] Campos LE, Pereira LF (2009). Pulmonary eosinophilia. J Bras Pneumol.

[5] Méndez-Sánchez N, Chávez-Tapia NC, Vazquez-Elizondo G, Uribe M (2007). Eosinophilic gastroenteritis: a review. Did Dis Sci.

[6] Grayson PC, Ponte C, Suppiah R (2022). 2022 American College of Rheumatology/European Alliance of Associations for Rheumatology classification for eosinophilic granulomatosis with polyangiitis. Ann Rheum Dis.

[7] Lau RA, Bains R, Suraweera D (2017). A rare case of digital ischemia and gangrene in ANCA-associated vasculitis with review of the literature. Case Rep Rheumatol.

[8] Wathurapatha W, Rathnamali BGA, Dissanayake U (2021). Sensory-motor polyneuropathy and digital ischemia: a rare presentation of granulomatosis with polyangiitis. Case Rep Rheumatol.

